# Ultrahigh Interfacial Thermal Conductance across GaN/BAs Interfaces via Atomic‐Level Engineering

**DOI:** 10.1002/advs.77516

**Published:** 2026-10-12

**Authors:** Linfeng Yu, Hongkai Zhang, Haofeng Qin, Yi Zhang, Jinyuan Xu, Yiming Zhao, Dongliang Ding, Jianbin Xu, Sunmi Shin, Zhuhua Zhang, Guangzhao Qin

**Affiliations:** ^1^ State Key Laboratory of Mechanics and Control for Mechanical Structures, College of Aerospace Engineering Nanjing University of Aeronautics and Astronautics Nanjing People's Republic of China; ^2^ State Key Laboratory of Advanced Design and Manufacturing Technology For Vehicle, College of Mechanical and Vehicle Engineering Hunan University Changsha People's Republic of China; ^3^ Department of Mechanical Engineering National University of Singapore Singapore Singapore; ^4^ Department of Electronics Engineering The Chinese University of Hong Kong Hong Kong People's Republic of China; ^5^ Greater Bay Area Institute for Innovation Hunan University Guangzhou Guangdong Province People's Republic of China; ^6^ State Key Laboratory of Materials for Integrated Circuits, Shanghai Institute of Microsystem and Information Technology Chinese Academy of Sciences Shanghai People's Republic of China

**Keywords:** atomic‐level engineering, GaN/BAs heterojunction, interfacial thermal conductance, machine learning potential, multiscale simulation

## Abstract

The integration of gallium nitride (GaN) with ultrahigh‐thermal‐conductivity boron arsenide (BAs) substrates represents a promising pathway for developing next‐generation high‐power devices. The heat dissipation capability of such integrated systems is fundamentally governed by thermal transport across GaN/BAs interfaces, and buffer layers such as Al_2_O_3_ have been widely adopted to accommodate lattice mismatch and facilitate heterointegration. However, the atomic‐scale structural factors governing thermal transport across GaN/BAs interfaces remain unclear, limiting further improvements in interfacial thermal conductance. Herein, we propose an atomic‐scale interfacial structural‐uniformity strategy for engineering ultrahigh thermal conductance across GaN/BAs interfaces using molecular dynamics simulations augmented by machine‐learning interatomic potentials. We show that interfacial structural uniformity plays a more important role than individual bond strength in determining thermal transport across the investigated GaN/BAs interfaces. For instance, the As‐N‐terminated coherent interface exhibits the highest interfacial structural uniformity and achieves an ultrahigh interfacial thermal conductance of approximately 2000 MW·m^−^^2^·K^−^^1^, representing a nearly eightfold enhancement over the experimentally reported Al_2_O_3_‐mediated interface (250 MW·m^−2^·K^−1^). Finite‐element analysis indicates that this atomic‐scale optimization directly translates into device‐scale performance, leading to a substantial improvement in overall heat dissipation efficiency. These findings suggest that engineering interfacial structural uniformity offers a promising strategy for achieving high‐efficiency thermal management in next‐generation high‐power electronic devices.

## Graphical abstract

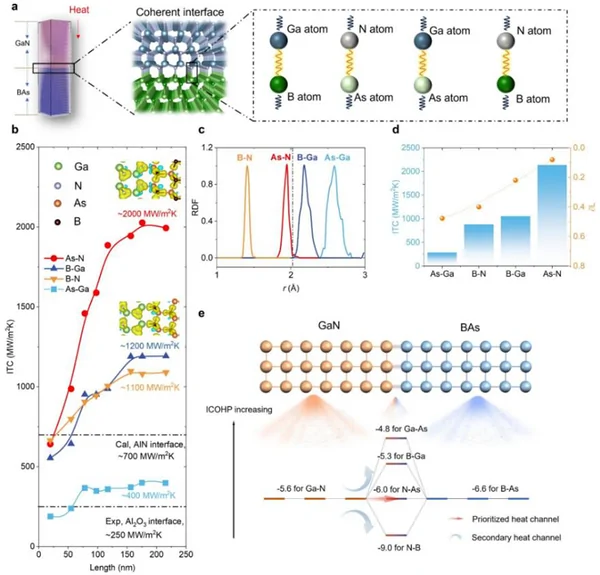

## 1 Introduction

The escalating miniaturization and integration of microelectronic devices have intensified localized thermal loads, driving the development of advanced thermal management strategies to ensure device reliability under extreme operating conditions [1, 2]. Although substrates with ultrahigh bulk thermal conductivity are essential for efficient heat extraction, it is interfacial thermal resistance, rather than bulk conductivity, that has emerged as the dominant bottleneck limiting heat dissipation efficiency in state‐of‐the‐art electronic systems. This bottleneck is especially acute in gallium nitride (GaN) devices, which are critical to 5G infrastructure and automotive power electronics and operate at power densities exceeding 5 kW cm^−2^, demanding highly efficient thermal management solutions [3]. Among representative substrate materials, including diamond, SiC, Si, AlN, Al_2_O_3_, SiO_2_, ZnO, Al, Au, Cr, and boron arsenide (BAs), only diamond and cubic BAs exhibit intrinsic thermal conductivities exceeding 1000 W m^−1^ K^−1^, as shown in Figure 1a. Yet diamond's severe lattice mismatch with GaN limits its interfacial thermal conductance to below 200 MW m^−2^ K^−1^, while other high‐κ candidates such as graphite and graphene face integration challenges [4, 5], and cubic boron nitride requires costly isotopic enrichment [6] without fully resolving compatibility limitations. Cubic BAs (Figure 1b) therefore stands out as the most viable substrate, combining an ultrahigh thermal conductivity (∼1300 W m^−1^ K^−1^, second only to diamond) with high carrier mobility [7, 8, 9, 10, 11, 12], and positioning it as a promising platform for next‐generation high‐power GaN devices [13]. However, this bulk advantage alone cannot guarantee efficient heat dissipation, because the overall thermal performance of GaN/BAs heterostructures is ultimately governed by the interface, making interfacial engineering the decisive challenge for realizing the full potential of BAs‐based thermal management.

**FIGURE 1:**
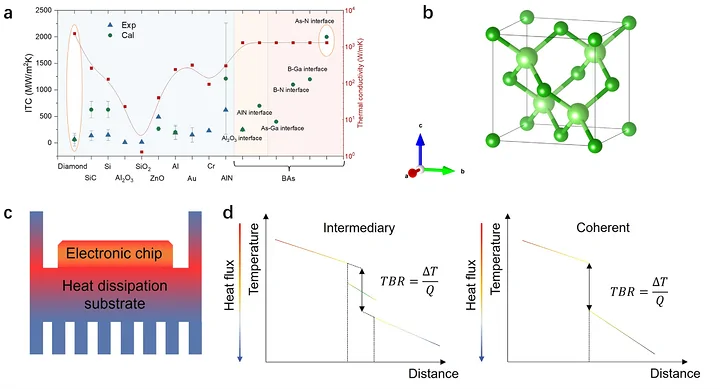
Interfacial thermal transport model of the GaN/BAs system. (a) Experimental and theoretical exploration of ITC and thermal conductivity; (b) Crystal structure of BAs; (c) Schematic diagram of chip and heat dissipation substrate; (d) Temperature gradient design for intermediary and coherent interfaces.

Two principal strategies are commonly used to improve interfacial thermal transport. The first introduces a buffer layer to form a transitional interface, alleviating lattice and thermal mismatches while maintaining acceptable heat transport; high‐κ interlayers are typically preferred to minimize interfacial resistance. For example, Kang et al. [13] achieved an ITC of approximately 250 MW m^−2^ K^−1^ in a GaN/BAs heterostructure using an Al_2_O_3_ interlayer, although the relatively low intrinsic thermal conductivity of Al_2_O_3_ and its moderate bonding strength with GaN constrain further improvement. The second, more effective strategy constructs direct buffer‐free interfaces, where efficient phonon transmission is promoted through optimized bonding configurations and lattice matching; because stronger interfacial bonding enhances electron‐phonon and phonon‐phonon coupling [14], such coherent interfaces offer a substantially higher theoretical ITC ceiling. Indeed, Wu et al. [15] predict that directly bonded GaN/BAs interfaces can reach ITC values as high as ∼500 MW m^−2^ K^−1^, nearly double that of buffer‐mediated interfaces [13]. Despite this progress, both strategies remain materials‐driven, focused on selecting or optimizing specific materials and interlayers rather than on a general design principle, so a systematic atomic‐level strategy for maximizing interfacial thermal transport remains lacking. Moving beyond conventional interface engineering toward deliberate atomic‐level design is therefore essential for fully exploiting the thermal transport potential of ultrahigh‐κ materials such as BAs.

Establishing such an atomic‐level design strategy is challenging because interfacial thermal transport spans multiple length scales, from interatomic bonding to device‐level heat dissipation. At the atomic scale, bonding strength and phonon dispersion determine intrinsic transport limits; at the nanoscale, defects, roughness, and interfacial morphology govern phonon scattering; and at the device scale, heat dissipation is controlled by temperature gradients and boundary conditions. Capturing these scale‐coupled phenomena is difficult for any single computational approach, and the available tools face corresponding limitations. Although first‐principles calculations provide quantum‐mechanical accuracy at the atomic scale, they are restricted to relatively small systems. Classical molecular dynamics (MD) simulations extend to larger, nanoscale systems but depend critically on the accuracy of the underlying interatomic potential. And finite‐element simulations (FES) capture device‐scale behavior but require reliable material parameters obtained from these lower‐scale models. These matched limitations highlight the need for an integrated multiscale framework capable of bridging quantum‐level accuracy, atomic‐scale resolution, and continuum‐scale predictability.

In this study, we employ this integrated multiscale framework to implement an atomic‐level engineering strategy, systematically investigating interfacial thermal transport in GaN/BAs heterojunctions. We first validate our model by reproducing experimental results for the Al_2_O_3_‐mediated interface, integrating machine learning potentials (MLP), first‐principles calculations, MD, and FES. We then design and engineer atomic‐scale interfaces for both buffer‐mediated and coherent types, quantitatively analyzing how atomic interfacial configurations regulate thermal transport. Specifically, we propose AlN as an alternative buffer layer through atomic‐scale phonon spectrum analysis, demonstrating its superior performance, and we examine the effects of different coherent bonding configurations on thermal transport efficiency at the atomic level, identifying an optimized interface. Finally, FES link these atomic‐scale insights to device‐level behavior, enabling quantitative comparisons of heat dissipation performance. This work establishes atomic‐scale design principles for interfacial thermal transport in GaN/BAs heterostructures, providing theoretical and engineering guidance for high‐performance thermal interfaces in next‐generation power devices.

## 2 Results and Discussion

### 2.1 Cross‐Scale and Interfacial Thermal Transport

The impetus for investigating ITC stems from the critical need to precisely control and enhance heat transfer across material interfaces in advanced devices. As semiconductor technology advances, the performance and reliability of these devices increasingly depend on effective management of ITC. The ITC is fundamentally governed by the vibrational characteristics of interfacial atoms, specifically phonons [17]. The ability of phonons to transfer heat is a key determinant of ITC. Typically, ITC is defined as the heat flux per unit area transmitted through an interface under a temperature gradient [18, 19]. However, this property can exhibit significant variability depending on the atomic and structural characteristics of the interface. When a transition layer is present, for instance, the thermal transport behavior becomes more complex due to nonuniform temperature gradients, often deviating substantially from that of the constituent materials. As illustrated in Figure 1c,d, to enable a systematic comparison of thermal conductance across different interface types, we define an effective ITC for both transition‐layer and coherent interfaces and construct detailed microscopic schematic models to represent these interfaces.

To gain an in‐depth understanding of and accurately predict the complex phonon‐mediated thermal transport at heterogeneous interfaces, we employ a comprehensive multiscale simulation framework, as shown in Figure 1a. First, at the atomic scale, we perform first principles calculations to obtain accurate energies and atomic forces for GaN, BAs, and their heterostructures. Based on these results, we construct and optimize atomic‐scale structural models for the GaN/BAs heterointerfaces. Subsequently, leveraging these high‐fidelity quantum mechanical data, we employ machine‐learning methods to establish mapping relationships between atomic configurations and their corresponding energies and forces, thereby developing a highly accurate and efficient MLP tailored to this heterosystem. The MLP approach has demonstrated significant application value and influence in multiple fields [20, 21, 22, 23]. The trained potential is then used to conduct non‐equilibrium molecular dynamics (NEMD) [18] simulations on the optimized atomic‐scale heterointerface models. By directly simulating heat flux across the interfaces, we obtain ITC values that capture realistic structural characteristics. Finally, these material thermal transport properties derived from MD simulations are incorporated as key constitutive parameters into FES, enabling device‐level thermal simulations to analyze macroscopic heat flux distributions and temperature variations in integrated devices with complex heterointerfaces. This integrated multiscale framework serves as the computational engine of our atomic engineering approach, allowing us to design, evaluate, and predict interface performance from the atomic scale up to the operational device level.

### 2.2 Atomic‐Level Design of Interfaces

Interfaces are the major source of thermal resistance, where the interfacial thermal resistance is governed by the atomic configuration at the interface. The schematic phonon‐scattering behavior of the interface model is presented in Figure 2b. And as shown in Figure 2c, an intermediary interface does not involve direct contact between the two materials but rather connects them through an interlayer. Because atomically precise coherent interface engineering is still a notable experimental challenge, inserting a transition layer (recently termed a “phonon bridge”) has become a commonly adopted and scalable engineering strategy. For instance, Shen et al. [24] in situ induced an amorphous SiO_2_ interlayer at the Ga_2_O_3_/SiC interface, achieving a thermal boundary conductance as high as 162  MW·m^−2^·K^−1^, which is roughly nine times that of the untreated van der Waals interface. Zhan et al. [25] employed a scalable stacking‐cutting process to fabricate thermally conductive pads with arch‐shaped structures by mixing hexagonal boron nitride nanosheets with a viscoplastic polymer. Specifically for the BAs/GaN heterojunction, Kang et al. [13] employed aluminum oxide (Al_2_O_3_) as the interlayer material in their experiments. In such a configuration, the total thermal resistance arises from three components: the GaN/Al_2_O_3_ interfacial thermal resistance, the intrinsic thermal resistance of Al_2_O_3_, and the BAs/Al_2_O_3_ interfacial thermal resistance. While this design improves interfacial compatibility, it complicates the modulation of thermal conductance. In contrast, a coherent interface, as depicted in Figure 2d, represents an ideal scenario where GaN and BAs are in direct atomic contact with a one‐to‐one lattice correspondence. The interfacial atoms belong simultaneously to both crystal lattices, a configuration that is expected to markedly suppress phonon scattering. In simulations, given that the transition layer thickness is negligible compared to the dimensions of the heat source and sink, the effective thermal conductance of an intermediary interface is evaluated via linear extrapolation at the midpoint of the interlayer. For a coherent interface, it is obtained directly from linear extrapolation at the interface itself. This methodological difference originates from the fundamental structural distinction of whether an interlayer is present. These two archetypes define the concrete objectives of our atomic engineering strategy, which focuses on optimizing the intermediary buffer region in one case and crafting a perfectly coherent atomic junction in the other.

**FIGURE 2:**
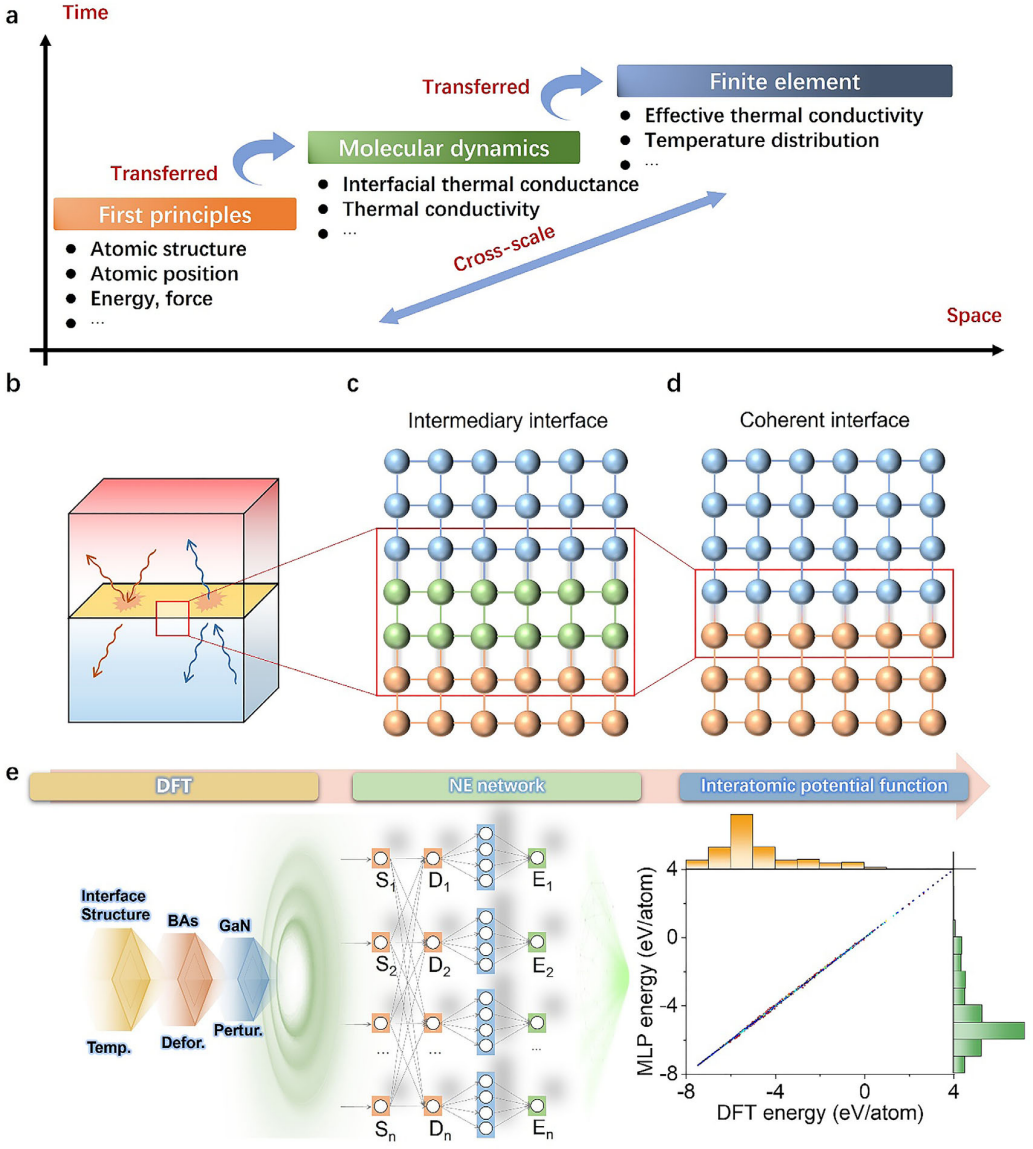
The multiscale interface design and simulation framework driven by MLP. (a) Cross‐scale workflow between first principles, molecular dynamics, and finite element methods; (b) Interface model and its atomic level of (c) intermediary and (d) coherent interfaces. (e) Workflow for constructing a MLP function model.

For multiscale simulations, the theoretical evaluation of ITC is essential to elucidate atomic scale heat conduction mechanisms. Among various methods, the non‐equilibrium molecular dynamics (NEMD) approach is widely used because it directly resolves the relationship between heat flux and temperature gradient. However, traditional simulations relying on empirical interatomic potentials face substantial limitations in describing complex interfacial interactions, which can lead to deviations from experimental results or first principles predictions. To overcome these challenges and improve both accuracy and reliability, this study employs a machine learning potential (MLP) approach. This approach has demonstrated high accuracy and robustness in complex systems [26, 27], particularly for heterojunction interfaces with strong nonlinear interactions, thereby enabling high fidelity simulations of ITC.

As shown in Figure 2e, we first perform high precision density functional theory (DFT) calculations to obtain the energies and forces of BAs, GaN, and their interfacial structures across diverse atomic configurations. This process involves systematic structural perturbations, lattice distortions, and finite temperature geometric optimizations. These steps collectively sample the atomic configuration space comprehensively, constructing a representative training dataset and providing reliable reference data for MLP training. Subsequently, a neural network based potential optimized via evolutionary strategies is developed. This potential effectively embeds the first principles potential energy surface into the molecular dynamics framework, thereby bridging DFT level accuracy with the efficiency of molecular dynamics simulations. Compared to conventional empirical potentials, the developed MLP achieves significantly improved prediction accuracy for energies and forces and demonstrates superior stability and generalization capability in thermal transport simulations.

### 2.3 Experimental Benchmark Test with Al_2_O_3_ Transition layer

To verify the accuracy and reliability of our study, we constructed a theoretical model of the GaN/BAs heterojunction to benchmark against experimental data. In the reported experiments, this heterojunction incorporated an Al_2_O_3_ transition layer approximately 2 nm thick [13]. To accurately replicate this structure, a three‐layer heterojunction model with a total length of ∼50 nm was built, consisting of ∼26 nm of GaN, ∼2 nm of Al_2_O_3_, and ∼26 nm of BAs. We then employed non‐equilibrium molecular dynamics (NEMD) simulations to investigate the thermal transport properties of this model at room temperature. Figure 3a shows the simulated temperature gradient distribution, where the red region denotes the GaN heat source, the light blue region represents the Al_2_O_3_ transition layer, and the dark blue region indicates the BAs heat sink. The resulting distribution exhibits a distinct transitional profile compared to a directly bonded interface, indicating that the Al_2_O_3_ layer significantly modulates the thermal conduction process.

**FIGURE 3:**
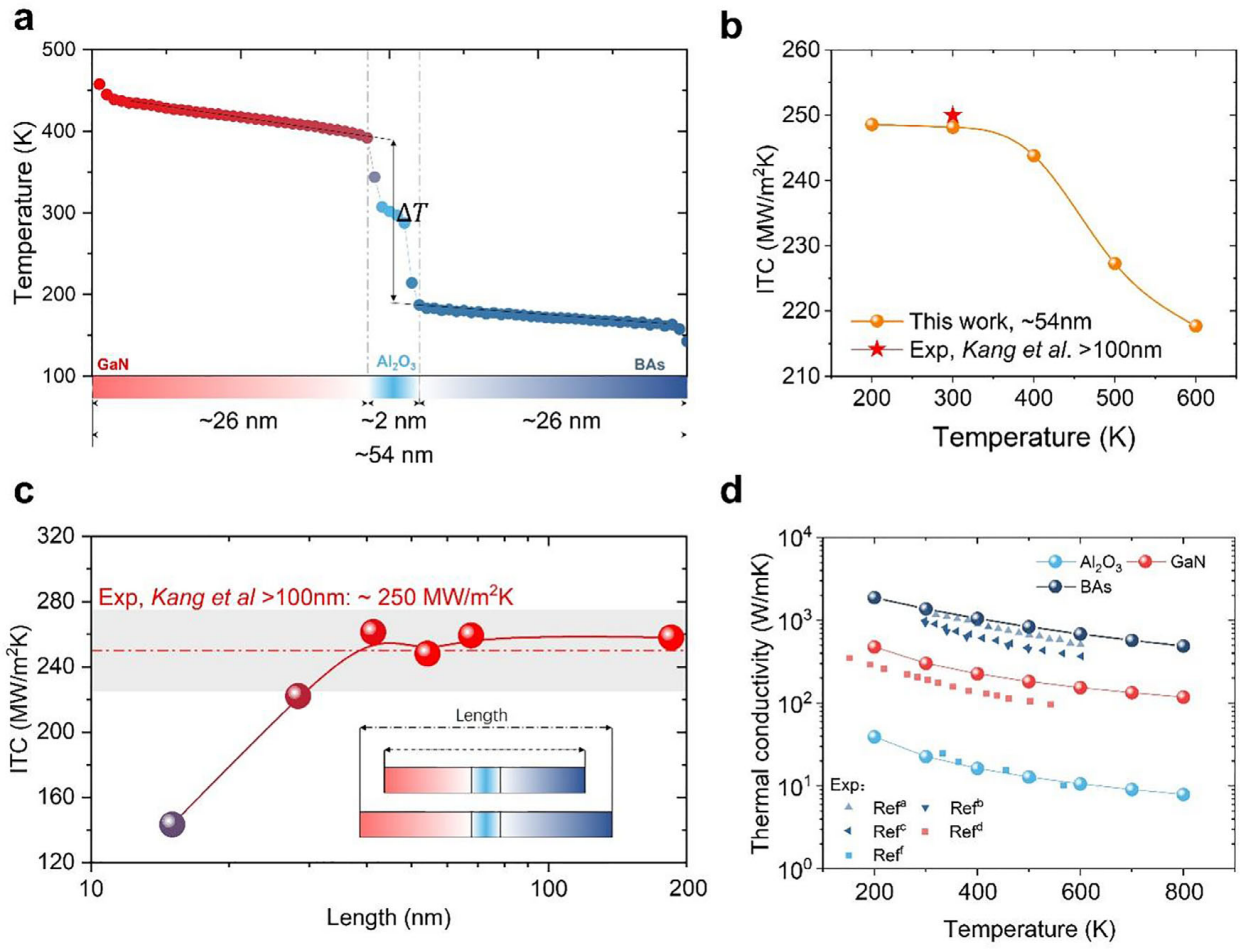
Benchmark of Al_2_O_3_ intermediary interface. (a) Thermal gradient of the Al_2_O_3_ intermediary interface model after NEMD simulation. (b) Temperature effect and (c) size effect of ITC of Al_2_O_3_ intermediate interface model based on NEMD simulation. (d) Intrinsic lattice thermal conductivity of Al_2_O_3_, GaN and BAs based on solving Boltzmann transport equation method. Ref^a,b,c^ [7, 11, 12], Ref^d^ [28], Ref^f^ [29].

We further evaluate the ITC of the model across a range of temperatures, as shown in Figure 3b. The results show a slight decrease in conductance with increasing temperature. Notably, our calculated room‐temperature ITC (∼248 MW m^−2^ K^−1^) closely matches the experimental value (∼250 MW m^−2^ K^−1^) reported by Kang et al. [13], thereby validating the accuracy and reliability of our theoretical model. Considering that the experimental GaN and BAs layers typically exceed 100 nm, we further investigated the effect of the transition layer material on phonon transport. As shown in Figure 3c, with the Al_2_O_3_ thickness fixed at 2 nm, we incrementally increased the GaN and BAs thicknesses. The simulation results agree well with the experimental data once the total thickness reaches 40 nm. Even beyond 100 nm, the deviation between simulation and experiment remains below 10% (gray region), highlighting the robustness and reliability of our simulation method across a wide range of device scales. In comparison, Wu et al. [15] recently investigated BAs/GaN interfacial thermal transport using deep‐learning potential functions but did not include the actual Al_2_O_3_ transition layer or other interface configurations in their model. Consequently, their size‐converged ITC (∼500 MW m^−2^ K^−1^) exceeded the experimental value by more than 200%. This successful benchmark validates our multiscale framework and, more importantly, establishes a credible foundation for the subsequent active design and engineering of interfaces, which is the core of our atomic engineering paradigm. In addition, quantum effects on interfacial heat transport were taken into account to validate our results, as presented in Note S1, Figure S1 and S2.

### 2.4 Enhanced Phonon Matching with AlN Transition Layer

Although the theoretical and experimental results show high consistency and both indicate exceptionally high ITC, a critical question remains whether Al_2_O_3_, when used as a transition layer, can fully unlock the thermal management potential of the GaN/BAs heterostructure. The core issue lies in the relatively low intrinsic thermal conductivity of Al_2_O_3_, which is much lower than that of GaN and BAs, leading to a severe mismatch in their atomic vibrational properties. As shown in Figure 3d, our calculated thermal conductivities for Al_2_O_3_, GaN, and BAs are approximately 20, 250, and 1300 W·m^−1^·K^−1^, respectively, all in good agreement with experimental data. Clearly, Al_2_O_3_ is at least an order of magnitude less conductive than GaN or BAs, reflecting its considerably poorer heat transfer capability. This significant difference suggests a strong mismatch in phonon properties between Al_2_O_3_ and the GaN/BAs system. Moreover, the ITC of the GaN/Al_2_O_3_ interface is relatively low [Figure 1a], which can become a bottleneck for overall heat dissipation. Although Al_2_O_3_ offers advantages such as lattice buffering and improved adhesion, its low thermal conductivity and limited ITC hinder efficient heat transfer, potentially leading to uneven heat distribution and degraded thermal performance of the GaN/BAs heterostructure. Thus, while Al_2_O_3_ can improve lattice matching and reduce interfacial stress, its thermal limitations may restrict the practical effectiveness of GaN/BAs thermal management. This motivates the search for alternative transition layer materials to enhance heat dissipation performance.

Given this context, we selected AlN as the transition layer due to its lattice compatibility with both GaN and BAs and its relatively high thermal conductivity. Notably, AlN has already been used experimentally to form GaN/AlN heterojunctions with high ITC, as shown in Figure 1a. As presented in Figure 4a, we calculated the interfacial thermal transport properties of GaN/AlN/BAs heterojunctions of various sizes while keeping the AlN thickness fixed at 2 nm, and directly compared the results with the Al_2_O_3_ case. The results show that the ITC of the AlN based interface converges to approximately 700 MW m^−2^ K^−1^, nearly three times higher than that of the Al_2_O_3_ based interface. To understand the underlying mechanism, we calculated the phonon dispersion relations of GaN, AlN, Al_2_O_3_, and BAs using the finite displacement method. According to the phonon mismatch model, ITC depends on the matching of phonon spectra and density of states (DOS). Good phonon matching promotes transmission, whereas large mismatches increase reflection and scattering, thereby reducing ITC. As shown in Figure 4b, AlN achieves better matching with the phonon spectra and DOS of both GaN and BAs, which reduces phonon scattering and reflection and thus enhances ITC. Further analysis of mode‐resolved phonon scattering rates and mean free paths, shown in Figures 4c,d, confirms that AlN exhibits phonon properties more consistent with GaN and BAs, enabling more efficient interfacial transport. Compared with Al_2_O_3_, AlN offers superior phonon compatibility, which leads to significantly improved interfacial heat transfer and overall thermal management performance of the GaN/BAs heterostructure. Therefore, by employing atomic‐scale phonon spectrum analysis, we have identified and validated AlN as a considerably superior buffer material, demonstrating how targeted atomic‐level property matching can drastically improve interfacial thermal transport.

**FIGURE 4:**
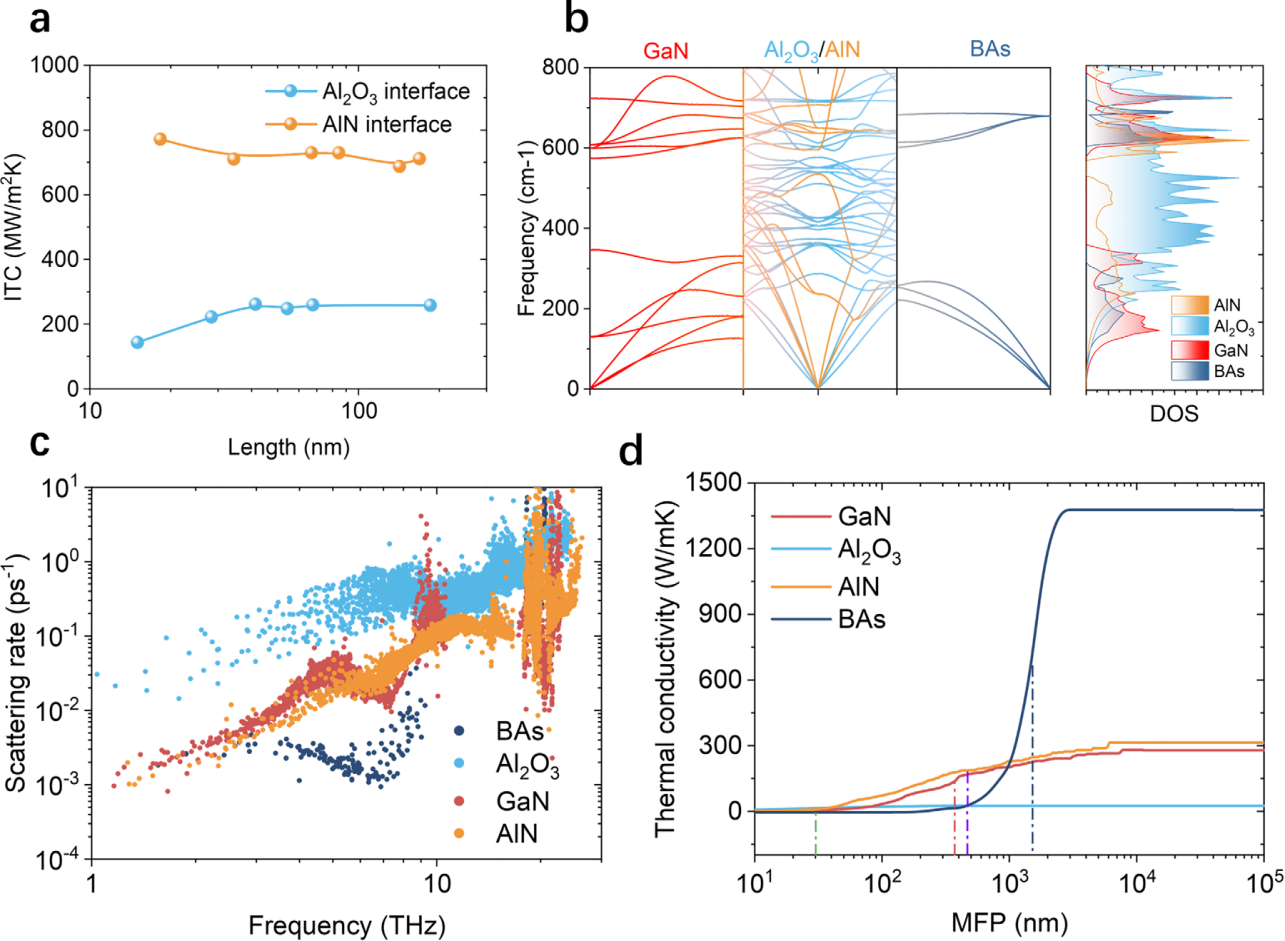
Phonon matching enhancement of AlN intermediary interface. (a) Comparison of ITC between AlN and Al_2_O_3_ intermediary interface after NEMD simulation. (b) Phonon dispersion and phonon state density of GaN, Al_2_O_3_/AlN, and BAs. (c) Comparison of the matching of intrinsic phonon scattering rates of GaN, Al_2_O_3_/AlN, and BAs. (d) Relationship between intrinsic cumulative thermal conductivity and phonon mean free path of GaN, Al_2_O_3_/AlN, and BAs.

### 2.5 Ultrahigh ITC in Coherent Interfaces

While optimizing the intermediary layer significantly boosts ITC, the ultimate thermal performance may be achieved by eliminating the interlayer altogether through the formation of coherent interfaces. The regulatory effect of aluminum nitride (AlN) as a transition layer on ITC underscores the critical importance of optimizing interlayer materials for thermal management. In practice, the effectiveness of such layers is often limited by lattice mismatch between the constituent materials. Lattice mismatch can introduce interfacial defects and stress concentrations, which subsequently degrade both ITC and overall thermal performance. This naturally leads to a key question how the GaN/BAs heterostructure can achieve its theoretical maximum ITC. Both GaN and BAs feature hexagonal honeycomb lattices similar to that of graphene and possess comparable atomic masses. To construct a commensurate interface, we transformed the cubic BAs (c‐BAs) primitive cell into a hexagonal lattice (without altering the actual atomic structure) via a suitable transformation matrix, yielding an in‐plane lattice constant of 3.40 Å, whereas hexagonal GaN (h‐GaN) exhibits a lattice constant of 3.25 Å. For the heterostructure, a reference lattice constant of 3.33 Å leads to mismatches of approximately 3% and 1.7% for *c*‐BAs and *h*‐GaN, respectively. The overall lattice mismatch therefore remains below 5%, which can be accommodated by coherent strain in thin‐film epitaxy under carefully selected crystallographic orientations. This structural similarity theoretically permits the formation of high conductance coherent interfaces, as illustrated in Figure 5a. It should be emphasized that such an ideal coherent interface relies on specific epitaxial alignment. Experimentally, it may be approached through strain‐compensated superlattices, low‐temperature nucleation layers, or similar strategies [30, 31] although it remains challenging to achieve defect‐free interfaces at large scales.

**FIGURE 5:**
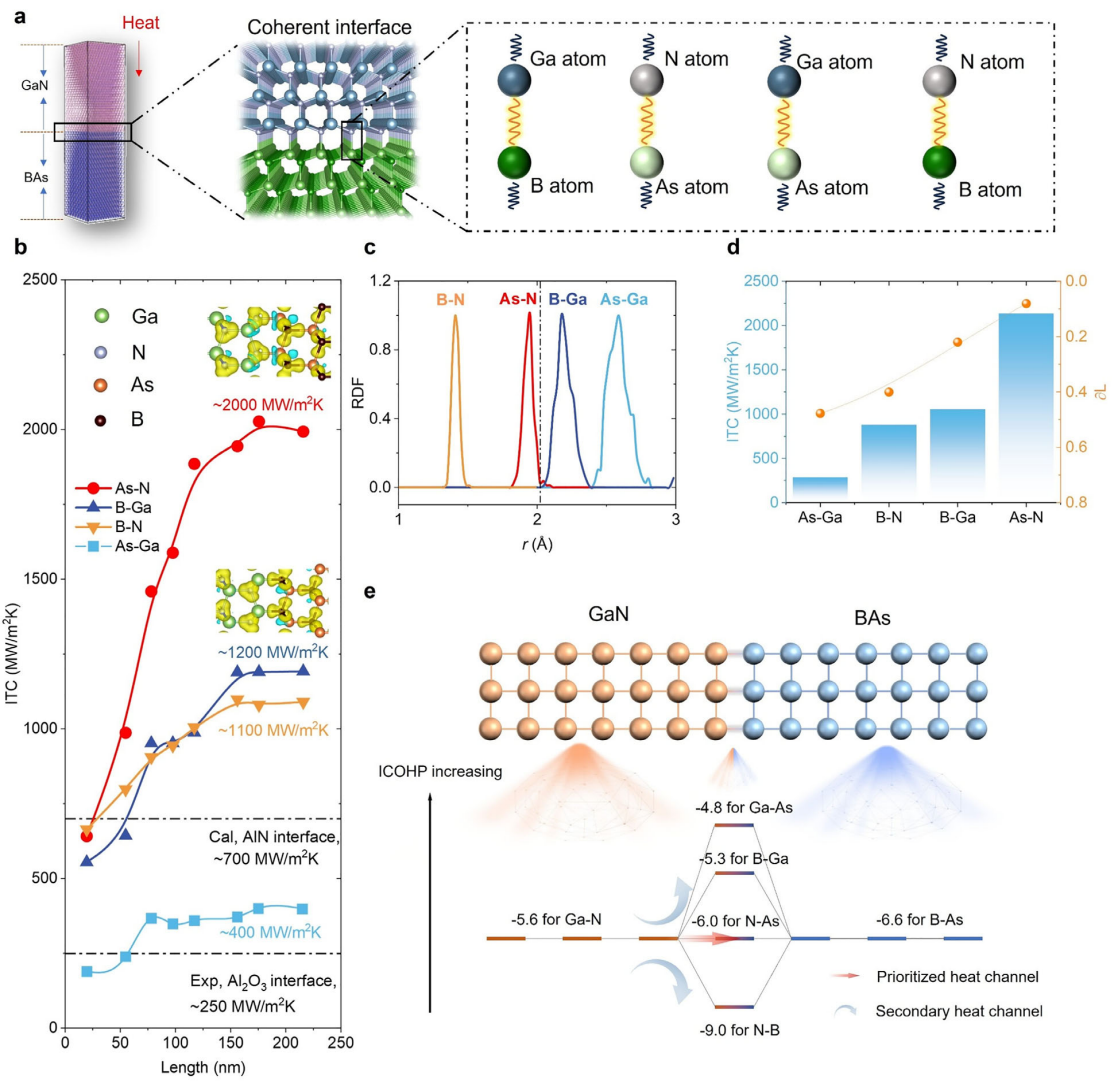
Atomic‐level engineering coherent interface of GaN/BAs. (a) Interface diagram of four different bonding methods. (b) Size effect of ITC for different coherent interfaces. (c) Radial distribution function after NEMD simulation at 300K. (d) The relationship between interface thermal conductance and bond length parameters at 300K. (e) Schematic diagram of the effect of interface uniformity on interface thermal conductance.

To test this hypothesis, we constructed four distinct directly bonded GaN/BAs interface models (Ga‐B, N‐As, Ga‐As, and N‐B) to evaluate the influence of atomic configuration and interfacial stress on thermal conductance. These coherent interfaces are characterized by continuous, ordered atomic arrangements with strong chemical bonding. Systematic size‐dependence analysis was performed to address the finite‐size effect caused by long phonon mean free paths (MFPs) in GaN and BAs, which reach the sub‐micro to micro scale as shown in Figure S2. Size‐dependent ITC calculations indicate that when the model size reaches ∼200 nm, the ITC converges with the following order: As‐N > B‐Ga ≈ B‐N > As‐Ga, as shown in Figure 5b. In the range of 0–100 nm, the ITC increases markedly with system size, reflecting the expected finite‐size effect. Crucially, when the cell size exceeds ∼200 nm, the ITC converges and remains nearly constant. This convergence length is substantially shorter than the bulk phonon MFPs because the interface itself acts as the dominant scattering source, compressing the size‐sensitive non‐equilibrium region into a narrow zone adjacent to the junction. Consequently, the non‐equilibrium phonon distribution near the interface reaches a steady state at a length scale far below the bulk MFP, validating that the cell sizes adopted in our MD simulations lie well within the converged regime.

The As‐N coherent interface exhibits the highest ITC, approximately 2000 MW m^−2^ K^−1^, a value more than eight times greater than typical experimental measurements. At the microscopic level, stronger interaction forces between atoms or molecules generally promote better thermal transport [14, 32, 33]. Specifically, the thermal conductance of covalent bond systems is usually higher than that of systems involving hydrogen bonds or van der Waals interactions. In principle, stronger interfacial bonding leads to higher ITC, and bond strength is often correlated with bond length, where shorter bonds typically indicate greater strength. However, our radial distribution function (RDF) analysis [34], presented in Figure 5c, reveals that the bond lengths for As‐N, B‐Ga, B‐N, and As‐Ga are 1.96, 2.14, 1.58, and 2.50 Å, respectively. Following traditional reasoning, the B‐N bond, being the shortest, should correspond to the highest ITC. Yet, the As‐N interface achieves the maximum ITC, a result that challenges the simplistic “bond‐strength‐dominant” assumption.

### 2.6 Quantification and Electronic Origin of Interface Uniformity

To characterize the deviation between the interfacial and bulk regions, we introduce a relative deviation parameter, denoted as *δX*. Consider a system consisting of one interfacial (transition) region and *n* bulk reference regions, each containing *m* measurable values of a given physical quantity *X*. The definition of *δX* follows a two‐step averaging process. For the *i*‐th bulk reference region, the average value of the physical quantity (denoted as 〈*X*_ref,*i*_〉) is calculated as

(1) $$\langle X_{\mathrm{ref},i}\rangle =\frac{1}{m}\sum_{j=1}^{m}X_{\mathrm{ref},i,j},\left(i=1,2,\text{…},n\right),$$

where *X*_ref,*i*, *j*_ represents the *j*‐th measurement of *X* in the *i*‐th bulk region, and *m* is the total number of such measurements. The overall average value for all bulk reference regions (denoted as 〈*X*_ref_〉_avg_) is then obtained by averaging over the *n* bulk regions:

(2)

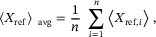




Based on the above definitions, the relative deviation parameter δX is defined as

(3) $$\delta X=\left|\frac{\langle X_{\mathrm{trans}}\rangle -{\langle X_{\mathrm{ref}}\rangle }_{\mathrm{avg}}}{{\langle X_{\mathrm{ref}}\rangle }_{\mathrm{avg}}}\right|,$$

where *X* represents any measurable physical quantity (e.g., bond length, energy, or density), and 〈*X*_trans_〉 denotes the average value of *X*within the interfacial region, obtained in the same way as for the bulk regions. When the system contains two bulk reference regions (*n* = 2) and *X* represents the bond length, the overall bulk average becomes the arithmetic mean of the two bulk bond lengths:

(4) $${\langle L_{\mathrm{ref}}\;\rangle }_{\mathrm{avg}}=\frac{\langle L_{\mathrm{bulk},1}\rangle +\langle L_{\mathrm{bulk},2}\rangle }{2}\;,$$

Substituting this into equation (3) gives

(5) $$\begin{array}{c} \delta L=|\frac{\left\langle L_{\mathrm{int}}\right\rangle -{\left\langle L_{\mathrm{ref}}\right\rangle }_{\mathrm{avg}}}{{\left\langle L_{\mathrm{ref}}\right\rangle }_{\mathrm{avg}}}|. \end{array}$$

By flexibly defining the measurable quantity *X* and the number of reference regions *n*, this generalized formulation provides a unified quantitative framework for describing a wide range of multiphase systems and physical scenarios. It enables a systematic assessment of the degree of consistency or deviation between interfacial and bulk regions.

For the GaN/BAs system, the average bond length along the heat‐flow direction was first obtained by averaging over the *m* bond lengths in the bulk regions, yielding 〈*L*_ref_ 〉_avg_ = 2.2 Å. A bond‐length deviation parameter (δL) was then introduced to quantify the correlation between interfacial structure and interfacial thermal conductance (ITC). Smaller δL values indicate higher structural consistency, whereas larger δL values signify greater structural mismatch. As shown in Figure 5d, δL exhibits a strong inverse correlation with ITC, indicating that structural uniformity at the interface is a key factor governing thermal transport. Mechanistically, a uniform interface minimizes phonon scattering because the interatomic interactions closely resemble those in the bulk, allowing phonons to traverse the interface with lower energy dissipation. In contrast, large δL values increase phonon scattering, shorten phonon mean free paths, and reduce ITC. To validate the influence of bond strength, we conducted an integrated crystal orbital Hamilton population (ICOHP) analysis [35, 36] as shown in Figure 5e. The B‐N bond exhibits the lowest ICOHP value, signifying the strongest bonding interaction; however, its ITC is not the highest because its bond strength deviates substantially from the bulk average. Conversely, the As‐N bond displays bonding interactions well matched with Ga‐N and B‐As bonds, enabling uniform phonon propagation paths and resulting in superior ITC.

In short, inhomogeneous interfacial bonding triggers complex phonon energy exchange and dissipation, increasing inelastic scattering and shortening phonon mean free paths, which ultimately lowers the interfacial thermal conductance. In contrast, coherent and uniform interfaces preserve consistency in bond length and bond strength, suppress inelastic scattering, and enhance phonon transmission efficiency. Therefore, structural uniformity at the atomic scale is a fundamental strategy for optimizing interfacial thermal transport in GaN/BAs heterostructures. Our work quantifies this principle and shows that achieving such uniformity through deliberate engineering of specific bonding configurations (As‐N) is the essence of successful atomic engineering for thermal management. The δL parameter provides a direct metric for guiding this design process. Notably, in the GaN/BAs system, the common zinc‐blende structure and nearly identical lattice constants enable an atomically coherent interface with uniform bonding, providing near‐ideal elastic phonon transmission channels. Uniformity in this context is realized at the atomic scale through a homogeneous interfacial bonding environment. Conversely, when two materials exhibit a substantial lattice or phonon density‐of‐states mismatch, an atomically abrupt interface can itself act as a barrier to phonon transport. As demonstrated by Liu et al. [37], controlled interfacial disorder can introduce an intermediate phonon density of states, thereby establishing an effective spectral uniformity that bridges the mismatched bulk vibrational modes. This disorder‐mediated uniformity opens additional inelastic transmission channels and consequently enhances interfacial thermal conductance. Both strategies thus share the common physical objective of achieving a seamless phonon transmission pathway, yet they apply to opposite extremes of intrinsic lattice and phonon spectrum matching.

### 2.7 Device‐Level Heat Dissipation

Finally, to bridge atomic‐scale interface engineering with the macroscopic heat dissipation requirements of devices, we integrate atomic‐level interface designs and mesoscopic interfacial thermal transport simulations as constitutive parameters in finite element modeling (FEM). More information regarding the multi‐scale coupling strategy can be found in Note S2. This enables a cross‐scale computational analysis of thermal transport in BAs/GaN heterointerfaces. As illustrated in Figure 6a, we constructed a stochastic polycrystalline FEM model with a bimodal grain size distribution while maintaining a 1:1 stoichiometric ratio between the BAs and GaN phases. Interfacial connectivity was explicitly engineered by incorporating three distinct thermal contact configurations, which reconstructed the realistic physical interface and established a robust geometric framework for subsequent thermal transport analysis. As shown in Figure 6b, steady state simulations revealed distinct temperature gradient distributions across randomly oriented grains under coherent interface conditions. High resolution mappings in Figure 6c–e delineate three interfacial architectures parameterized by molecular dynamics (MD) simulation: the Al_2_O_3_ intermediary interface, the AlN intermediary interface, and the coherent interface, with corresponding ITCs of 250, 700, and 2000 MW m^−2^ K^−1^.

**FIGURE 6:**
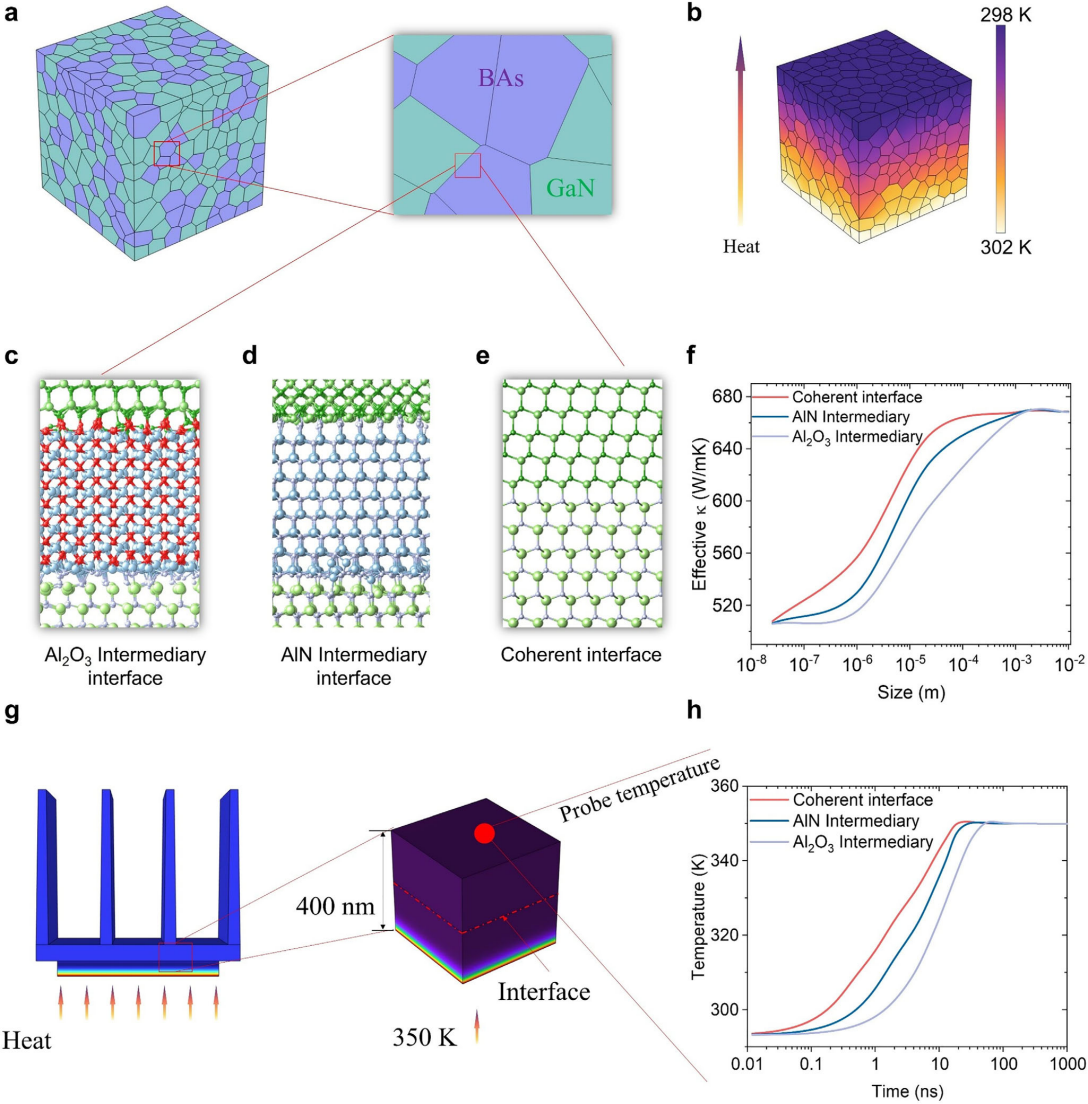
Multiscale simulations of thermal transport in GaN/BAs. (a) Finite element modelling of random grains; (b) Temperature distribution for one‐dimensional steady‐state heat transfer; (c) Al_2_O_3_ intermediary interface; (d) AlN intermediary interface; (e) Coherent interface; (f) Comparison of effective thermal conductivity with a finite model; (g) Finned radiator and corresponding simplified two‐layer finite element model; (h) Temperature changes at different interfaces.

As shown in Figure 6f, one‐dimensional steady state modeling revealed a size dependent correlation between ITC and effective thermal conductivity (*κ_eff_*) across the scale of 10^−7^ to 10^−4^ m. Within the submicron regime (10^−7^ to 10^−6^ m), ITC positively correlates with *κ_eff_*, indicating that higher ITC directly enhances overall heat transfer efficiency. In this regime, interfacial transport dominates thermal performance, controlling both the interfacial heat flux and the *κ_eff_* of the composite system. Beyond 10^−4^ m, *κ_eff_* becomes primarily governed by the intrinsic bulk conductivity, and the influence of ITC diminishes. This marks a transition from interface dominated to bulk dominated thermal transport, which is a characteristic size effect in multiscale heat transfer.

To further demonstrate device level implications, a simplified double layer interfacial model based on a finned heat sink geometry was constructed for transient FEM simulations, as shown in Figure 6g. The 400 nm scale model incorporated a 350 K heat source boundary, while the temperature evolution on the opposite surface was monitored in real time via a virtual nanoprobe. As shown in Figure 6h, within 100 ns, the coherent interface exhibited the highest temperature response and the fastest thermal relaxation, surpassing the Al_2_O_3_ and AlN configurations. This pronounced kinetic advantage confirms that atomically coherent lattice matching minimizes phonon scattering, accelerates heat flux propagation, and enables efficient device level thermal modulation and dissipation. Most significantly, it provides direct evidence at the device level that atomic engineering, implemented through our multiscale framework, can successfully translate atomic‐scale optimizations into tangible performance gains in realistic device geometries.

## 3 Conclusions

This study demonstrates the power of atomic engineering as a paradigm for mastering interfacial thermal transport. Using the GaN/BAs heterojunction as a model system, we constructed an integrated multiscale framework that serves as the enabling platform for this approach. By focusing on two distinct interface types, we executed a strategy of atomic‐scale design. First, through atomic‐scale phonon analysis, we identified and validated AlN as a superior alternative to Al_2_O_3_, increasing the interfacial thermal conductance to approximately 700 MW·m^−2^·K^−1^. More profoundly, by explicitly designing and evaluating different coherent atomic bonding configurations, we achieved an unprecedented interfacial thermal conductance of about 2000 MW·m^−2^·K^−1^ with an As‐N interface. We revealed that the underlying mechanism is structural uniformity at the atomic level, quantified by our *δL* parameter, which represents a core objective of the atomic engineering paradigm. Furthermore, device‐scale simulations confirmed that these atomically designed interfaces directly enhance macroscopic heat dissipation, with the As‐N coherent interface performing optimally. Beyond advancing the field from observation to active design, this work establishes an integrated multiscale simulation framework as a robust platform for atomic engineering.

## 4 Method

### 4.1 Machine Learning and Interatomic Potential Functions

The training dataset for the neuroevolution potential (NEP) was constructed by extensively sampling diverse atomic configurations of both bulk phases and heterogeneous interfaces. For the bulk systems, supercells containing 64 atoms (BAs), 32 atoms (GaN and AlN), and 30 atoms (Al_2_O_3_) were employed. Five distinct heterointerfaces critical to this study were explicitly modeled, namely BAs/GaN, BAs/Al_2_O_3_, BAs/AlN, GaN/AlN, and GaN/Al_2_O_3_, covering all interface configurations investigated in the subsequent thermal transport simulations. To comprehensively populate the configurational space and ensure the generalizability of the potential, two complementary sampling strategies were adopted. First, active‐learning‐driven ab initio molecular dynamics (AIMD) simulations were carried out in the NVT ensemble at temperatures of 300, 600, and 900 K, with each supercell concurrently subjected to uniaxial and biaxial strains ranging from −2% to +2%. Snapshots extracted from these trajectories inherently capture the coupled effects of thermal disorder and lattice deformation. Second, random atomic displacements with amplitudes of 0‐0.02 Å were applied to the fully relaxed configurations, in combination with additional random lattice strains, to further augment the dataset and improve its coverage of local atomic environments. By integrating these strategies, all reference energies and atomic forces for these structures were obtained from density functional theory (DFT) calculations performed with the Vienna Ab initio Simulation Package (VASP) [38] using the same parameters described in the previous subsection: the projector augmented wave (PAW) method with the Perdew‐Burke‐Ernzerhof (PBE) exchange‐correlation functional [39], a kinetic energy cutoff of 1000 eV for the plane‐wave basis, and energy and force convergence criteria of 10^−5^ eV and 10^−4^ eV·Å^−1^, respectively. More information regarding interface modeling and mismatch can be found in Note S3.

### 4.2 Molecular Dynamics Simulations

Based on the trained potential function, the ITC is further evaluated using NEMD simulations. The fundamental principle of this calculation involves establishing a temperature gradient across the system and determining the thermal conductance by analyzing the system's non‐equilibrium response. According to Fourier's law, the ITC is defined as:

(6) $$\begin{array}{c} ITC=\frac{Q}{\Delta T}\;, \end{array}$$

where *Q* represents the heat flux density across the interface, and Δ*T* is the temperature difference or gradient applied in the system. In this context, *Q* is computed from the heat flow within the simulation, while ΔT is derived from the imposed temperature gradient. This approach allows for the accurate determination of the ITC by capturing the response of the system to thermal perturbations.

### 4.3 Phonon Boltzmann Transport Equation

By solving the phonon BTE, the thermal conductivity can be obtained as [40, 41]:

(7) $$\kappa =\kappa_{\alpha \alpha }=\frac{1}{V}\;\sum_{\lambda }C_{\lambda }\nu_{\lambda \alpha }^{2}\tau_{\lambda \alpha },$$

where τ_λα_, *C*_λ_, ν_λα_, and *V* are the phonon relaxation time, specific heat capacity, group velocity for the λ mode phonon along the α direction, and the crystal volume, respectively. The IFCs can be obtained from finite difference calculations based on Taylor expansion of the potential energy *E* :

(8) $$\begin{array}{cc} E & =E_{0}+\frac{1}{2}\sum_{\begin{array}{c} ij\\ \alpha \beta \end{array}}\Phi_{ij}^{\alpha \beta }r_{i}^{\alpha }r_{j}^{\beta }+\frac{1}{3!}\sum_{\begin{array}{c} ijk\\ \alpha \beta \gamma \end{array}}\Phi_{ijk}^{\alpha \beta \gamma }r_{i}^{\alpha }r_{j}^{\beta }r_{k}^{\gamma }\\ & \;+\frac{1}{4!}\sum_{\begin{array}{c} ijkl\\ \alpha \beta \gamma \delta \end{array}}\Phi_{ijkl}^{\alpha \beta \gamma \delta }r_{i}^{\alpha }r_{j}^{\beta }r_{k}^{\gamma }r_{l}^{\delta }+\cdot \cdot \cdot , \end{array}$$

where the coefficients $\Phi_{ij}^{\alpha \beta }$, $\Phi_{ijk}^{\alpha \beta \gamma }$, and $\Phi_{ijkl}^{\alpha \beta \gamma \delta }$ correspond to the second‐order, third‐order, and fourth‐order force constants, respectively, and α, β, γ, δ and *i*, *j*, *k*, *l* are direction and atom indices respectively.

### 4.4 Finite Element Simulation

The core foundation of finite element heat conduction modeling lies in the classical Fourier's law and the principle of energy conservation, which are the two fundamental physical laws forming the theoretical cornerstone of all heat transfer analyses. When there are heat sources or heat sinks inside the material, the theoretical basis for the finite element numerical solution, whether for steady‐state heat transfer or transient heat transfer processes, can be uniformly expressed as the generalized heat conduction equation:

(9) $$\begin{array}{c} \rho c_{p}\left(\partial T/\partial t\right)+\nabla \cdot \left(-\kappa \nabla T\right)=Q, \end{array}$$

where *T* is the temperature, ρ is the density, *c_p_* is the specific heat capacity, κ is the thermal conductivity, and *Q* is the heat source term. For steady‐state heat transfer problems, the system has reached a state of thermal equilibrium, and the temperature field no longer changes with time. Therefore, the time derivative term ∂*T*/∂*t* = 0, and the governing equation is significantly simplified to an elliptic partial differential equation:

(10) $$\begin{array}{c} \nabla \cdot \left(-\kappa_{eff}\nabla T\right)=Q, \end{array}$$

## Author Contributions

G.Q. and Z.Z. S.S. supervised the project. L.Y. performed all the calculations, analysis and writing. All the authors contributed to interpreting the results. The manuscript was written by L.Y. with contributions from all the authors.

## Conflicts of Interest

The authors declare no conflicts of interest.

## Supporting information




**Supporting File**: advs77516‐sup‐0001‐SuppMat.docx.

## Data Availability

The data that support the findings of this study are available from the corresponding author on reasonable request.
