# Supplementary material for: Ultrahigh Interfacial Thermal Conductance across GaN/BAs Interfaces via Atomic‐Level Engineering
Source: Adv Sci (Weinh). 2026 Oct 12:e77516. Online ahead of print. doi: 10.1002/advs.77516 (PMC13658892; doi:10.1002/advs.77516)
Supplement: Supplementary file 1 [file ADVS-9999-e77516-s001.docx]

Ultrahigh Interfacial Thermal Conductance Across GaN/BAs Interfaces *via* Atomic-Level Engineering

Linfeng Yu^1,2,3^, Hongkai Zhang^2^, Haofeng Qin^2^, Yi Zhang^2^, Jinyuan Xu^2^, Yiming Zhao^3^, Dongliang Ding^4^, Jianbin Xu^4^, Sunmi Shin^3^,* Zhuhua Zhang^1^,* and Guangzhao Qin^2,5,6,*^

^1^ State Key Laboratory of Mechanics and Control for Mechanical Structures, College of Aerospace Engineering, Nanjing University of Aeronautics and Astronautics, Nanjing 210016, P. R. China

^2^ State Key Laboratory of Advanced Design and Manufacturing Technology for Vehicle, College of Mechanical and Vehicle Engineering, Hunan University, Changsha 410082, P. R. China

^3^ Department of Mechanical Engineering, National University of Singapore, Singapore, 117575 Singapore

^4^ Department of Electronics Engineering, The Chinese University of Hong Kong, Hong Kong, 999077 China

^5^ Greater Bay Area Institute for Innovation, Hunan University, Guangzhou 511300, Guangdong Province, China

^6^ State Key Laboratory of Materials for Integrated Circuits，Shanghai Institute of Microsystem and Information Technology，Chinese Academy of Sciences，865 Changning Road, Shanghai 200050 China

^*^ Corresponding author: [mpeshin@nus.edu.sg](mailto:mpeshin@nus.edu.sg) (S. S.), chuwazhang@nuaa.edu.cn (Z. Z.) and gzqin@hnu.edu.cn (G. Q.)

**Note S1. Quantum correction approach**

In nanoscale heat transfer, treating phonons as a classical gas under equipartition fails at temperatures below or around the Debye temperature, where quantum statistics dominate. This failure is stark in atomistic simulations like molecular dynamics: the classical phonon occupation imposes a frequency‑independent modal energy of *k_B_T*, whereas a real quantum oscillator stores far less energy at high frequencies. To bridge this gap, one applies a mode‑resolved quantum correction. The standard approach leverages the ratio of the quantum to classical heat capacity of a harmonic oscillator, which quantifies the mismatch in energy storage between the two statistics. For a single mode of angular frequency ω, the classical energy is *E_cl_=k_B_T*, giving a constant heat capacity *Gcl=∂E_cl_/∂T=k_B_*. The quantum energy (zero‑point excluded) is *E_q_*=ℏω[exp(ℏω/k_B_T)-1]−1, yielding the heat capacity [1]:

$$\begin{aligned} Gq=k_{B}\frac{u^{2}e^{u}}{\left( e^{u}-1 \right)^{2}}, u=\frac{\hbar\omega}{k_{B}T}.\#\left( 1 \right) \end{aligned}$$

Within the Landauer formalism, the classical spectral interfacial conductance *G_classic_*(ω) is proportional to *G_cl_*, the transmission function *T*(ω) and the group velocity. Assuming T(ω) the transmission to be temperature-independent and identical in classical and quantum descriptions to first order, the quantum-corrected spectral conductance obeys:

$$\begin{aligned} G_{QC}\left( w \right)=G_{classic}\left( w \right)\times\frac{Gq}{Gcl}=G_{classic}\left( w \right)\frac{u^{2}e^{u}}{\left( e^{u}-1 \right)^{2}}.\#\left( 2 \right) \end{aligned}$$

We applied this correction to the Al_2_O_3_ interlayer in a BAs/GaN heterostructure (model size $50.14\times52.12\times217.44$ nm^3^), following the experimental configuration of *Kang et al*.  [2]. Surprisingly, the frequency‑dependent interfacial conductance after quantum correction remained almost identical to the classical MD result (Fig. S1). This near‑invariance arises because the dominant heat‑carrying phonons in this system, at the simulation temperature, correspond to $u\ll1$. In that limit, the correction factor expands as$1+O(u^{2})$, staying exceedingly close to unity. Consequently, the classical simulation already delivers a physically reasonable estimate for this specific structure, and the quantum correction, while formally indispensable, introduces only a marginal quantitative change.

**Note S2. Multiscale coupling strategy**

A Voronoi-tessellated polycrystalline model generated by Neper provides the geometric basis for cross-scale mapping. The locally planar grain boundary segments share the same topology as the bicrystal flat interfaces used in molecular dynamics (MD) simulations. A three-level parameter transfer chain is constructed. At the atomic scale, density functional theory and machine-learned potentials yield size-independent interfacial thermal conductances (ITCs) for Al₂O₃-mediated, AlN-mediated, and coherent As-N interfaces via non-equilibrium MD. These ITCs incorporate bonding heterogeneity, phonon spectrum mismatch, and defect scattering. At the mesoscale, a one-dimensional steady-state model computes size-dependent effective thermal conductivity from 100 nm to 100 μm, distinguishing the interface-dominated regime below 1 μm and the bulk-dominated regime above 100 μm. At the device scale, finite element simulations performed with COMSOL assign the reciprocal of the MD-derived ITC as interfacial thermal resistance to each Voronoi grain boundary. Bulk regions adopt experimental or first-principles intrinsic conductivities. Size effects are inherently embedded through the ratio of mesh characteristic size to thermal diffusion length without additional post-correction. From the perspective of finite element homogenization, when the device characteristic length far exceeds the atomic spacing, atomic-scale interface complexities can be homogenized into a single scalar interfacial thermal resistance. This enables self-consistent mapping from discrete atomic systems to continuum interface units, a strategy validated in prior work [3]. Two simplifications are adopted. First, a uniform ITC is assigned to all grain boundaries, neglecting misorientation dependence. This assumption is explicitly stated in the revised manuscript, and a misorientation-dependent database is planned for future work. Second, Fourier’s law is used as the governing equation, which does not fully describe pure ballistic transport. The minimum finite-element model size is 400 nm, which lies in the ballistic-diffusive transition regime. The performance ranking of coherent, AlN-mediated, and Al₂O₃-mediated interfaces is determined by the intrinsic phonon transport properties of the interfaces, unaffected by these simplifications, and qualitatively verified by all-atom MD under consistent conditions. All key thermal transport parameters originate from the same atomic-level theoretical framework. Model limitations are stated in the Conclusion section of the revised manuscript.

**Note S3. Interface Modeling and Matching**

The significant lattice mismatch between GaN and BAs is an important factor governing interfacial thermal transport, and we address it by considering two representative interface configurations that reflect distinct experimental realities. For the coherent interface, cubic BAs (c-BAs) and hexagonal GaN (h-GaN) share similar honeycomb-like atomic arrangements and comparable atomic masses. By transforming the c-BAs primitive cell into a hexagonal representation via the lattice matrix:

$$\begin{aligned} M=\left( \begin{matrix} 1 & -1 & 0 \\ 0 & 1 & 0 \\ 0 & 0 & 1 \end{matrix} \right),\#\left( 3 \right) \end{aligned}$$

the resulting in-plane lattice constant is 3.40 Å, while h-GaN exhibits a lattice constant of 3.25 Å. Adopting a reference lattice constant of 3.33 Å for the heterostructure, the mismatches for c-BAs and h-GaN are approximately 3% and 1.7%, respectively, yielding an overall mismatch below 5% that can be accommodated by coherent strain under carefully selected crystallographic orientations. Such an ideal coherent interface may be approached experimentally through strain-compensated superlattices or low-temperature nucleation layers, although it remains technically demanding [4,5]. For the intermediary interface, atomically precise interface control remains experimentally challenging, and a widely adopted strategy is to introduce a transition interlayer, often referred to as a phonon bridge. For instance, an amorphous SiO_2_ interlayer inserted at the Ga_2_O_3_/SiC interface has been shown to yield a thermal boundary conductance (TBC) as high as 162 MW m^-2^ K^-1^, approximately nine times that of the untreated van der Waals interface [6]. For the BAs/GaN system specifically, Kang et al. employed an Al₂O₃ interlayer and reported a TBC of 250 MW m^-2^ K^-1^ [2]. Our calculations for this configuration are in good agreement with these experimental values, validating the applicability of our modeling approach to engineering-relevant interfaces. These results collectively demonstrate that, while lattice-mismatch-induced strain and interfacial defects can in principle impede phonon transmission, appropriate interface engineering, whether through coherent epitaxial growth or deliberate interlayer insertion, can effectively regulate and enhance interfacial thermal transport in the BAs/GaN heterojunction.

**Figure S1. Comparison between classical molecular dynamics and the quantum correction approach.**

**Figure S2: Phonon-mean-free-path-dependent thermal conductivity.**

**References**

[1]	Y. Wang, L. Xie, H. Yang, M. Hu, X. Qian, R. Yang, and J. He, Strong Orbital-Lattice Coupling Induces Glassy Thermal Conductivity in High-Symmetry Single Crystal BaTiS 3, Phys. Rev. X **15**, 011066 (2025).

[2]	J. S. Kang, M. Li, H. Wu, H. Nguyen, T. Aoki, and Y. Hu, Integration of boron arsenide cooling substrates into gallium nitride devices, Nat Electron **4**, 416 (2021).

[3]	B. Mortazavi, M. Silani, E. V. Podryabinkin, T. Rabczuk, X. Zhuang, and A. V. Shapeev, First-Principles Multiscale Modeling of Mechanical Properties in Graphene/Borophene Heterostructures Empowered by Machine-Learning Interatomic Potentials, Advanced Materials **33**, 2102807 (2021).

[4]	M. A. Moram, C. S. Ghedia, D. V. S. Rao, J. S. Barnard, Y. Zhang, M. J. Kappers, and C. J. Humphreys, On the origin of threading dislocations in GaN films, J. Appl. Phys. **106**, 073513 (2009).

[5]	T. Kehagias et al., Nanostructure and strain in InGaN/GaN superlattices grown in GaN nanowires, Nanotechnology **24**, 435702 (2013).

[6]	Y. Shen et al., Improving interfacial thermal conductivity by constructing covalent bond between Ga₂O₃ and SiC, Nat Commun **16**, 10723 (2025).
